# Genetic differentiation in the *MAT*-proximal region is not sufficient for suppressing recombination in *Podospora anserina*

**DOI:** 10.1093/g3journal/jkaf015

**Published:** 2025-01-24

**Authors:** Pierre Grognet, Robert Debuchy, Tatiana Giraud

**Affiliations:** CEA, CNRS, Institute for Integrative Biology of the Cell, Université Paris-Saclay, Gif-sur-Yvette 91198, France; CEA, CNRS, Institute for Integrative Biology of the Cell, Université Paris-Saclay, Gif-sur-Yvette 91198, France; Ecologie Systematique et Evolution, CNRS, Université Paris-Saclay, AgroParisTech, Gif-sur-Yvette 91198, France

**Keywords:** fungi, recombination suppression, sex chromosomes, mating-type chromosomes, mutant, neutral model

## Abstract

Recombination is advantageous over the long term, as it allows efficient selection and purging deleterious mutations. Nevertheless, recombination suppression has repeatedly evolved in sex- and mating-type chromosomes. The evolutionary causes for recombination suppression and the proximal mechanisms preventing crossing overs are poorly understood. Several hypotheses have recently been suggested based on theoretical models, and in particular that divergence could accumulate neutrally around a sex-determining region and reduce recombination rates, a self-reinforcing process that could foster progressive extension of recombination suppression. We used the ascomycete fungus *Podospora anserina* for investigating these questions: a 0.8-Mbp region around its mating-type locus is nonrecombining, despite being collinear between the 2 mating types. This fungus is mostly selfing, resulting in highly homozygous individuals, except in the nonrecombining region around the mating-type locus that displays differentiation between mating types. Here, we test the hypothesis that sequence divergence alone is responsible for recombination cessation. We replaced the *mat−* idiomorph by the sequence of the *mat+* idiomorph, to obtain a strain that is sexually compatible with the *mat−* reference strain and isogenic to this strain in the *MAT*-proximal region. Crosses showed that recombination was still suppressed in the *MAT*-proximal region in the mutant strains, indicating that other proximal mechanisms than inversions or mere sequence divergence are responsible for recombination suppression in this fungus. This finding suggests that selective mechanisms likely acted for suppressing recombination, or the spread of epigenetic marks, as the neutral model based on mere nucleotide divergence does not seem to hold in *P. anserina*.

## Introduction

Recombination is widespread in eukaryotes, as it is advantageous over the long term. Recombination breaks up allelic combinations, which allows more efficient selection and the purging of deleterious mutations (Muller 1932; Hill and Robertson 1966). Nevertheless, recombination can be suppressed locally in genomes, the most studied cases being on sex chromosomes (Ellegren 2000; Bachtrog 2014; Beukeboom and Perrin 2014; Cortez *et al.* 2014; Ma and Veltsos 2021). Recombination suppression has repeatedly evolved in sex chromosomes, although the reason why is still debated (Wright *et al.* 2016; Abbott *et al.* 2017; Ponnikas *et al.* 2018; Charlesworth 2021; Jay *et al.* 2024; Saunders and Muyle 2024). Indeed, while sexual antagonism has long been a commonly accepted hypothesis to explain progressive extension of recombination cessation on sex chromosomes, for linking to sex-determining genes other genes with alleles beneficial in only 1 of the sexes (Charlesworth *et al.* 2005, 2021), little evidence could be found in favor of this hypothesis (Ironside 2010). Furthermore, repeated evolution of recombination suppression has also been shown on fungal mating-type chromosomes, despite the lack of sexual antagonism (Fraser *et al.* 2004; Menkis *et al.* 2008; Branco *et al.* 2017, 2018; Bazzicalupo *et al.* 2019; Hartmann, Ament-Velásquez, *et al.* 2021; Hartmann, Duhamel, *et al.* 2021; Duhamel *et al.* 2022; Vittorelli *et al.* 2023; Jay *et al.* 2024). Indeed, there are no sex roles, or other obvious differentiated traits, associated with mating types in fungi and that would be controlled by genes distinct from the mating-type genes themselves (Branco *et al.* 2017; Bazzicalupo *et al.* 2019; Hartmann, Duhamel, *et al.* 2021).

Other hypotheses than sexual antagonism have therefore been proposed to explain the evolution of recombination suppression on sex-related chromosomes (Wright *et al.* 2016; Abbott *et al.* 2017; Kent *et al.* 2017; Ponnikas *et al.* 2018; Jeffries *et al.* 2021; Jay *et al.* 2022, 2024; Saunders and Muyle 2024). It has been suggested that there could be a selection of nonrecombining fragments that would carry fewer deleterious mutations than average, and that the few recessive deleterious mutations would be sheltered at the heterozygous stage when associated with a Y-like sex-determining or mating-type determining gene, allowing the fixation of nonrecombining fragments despite their load (Jay *et al.* 2022, 2024; Lenormand and Roze 2022). This would correspond to an evolutionary cause selecting against recombination, while the proximal mechanism preventing recombination could be inversions or any other mechanism, from *cis*- or *trans*-acting recombination modifiers to epigenetic marks. Another hypothesis involves recombination-suppressing epigenetic marks, possibly associated with transposable elements for their silencing, known to accumulate in nonrecombining regions, and that could spread in nearby regions (Kent *et al.* 2017). A related hypothesis postulates that sequences accumulate differences between sex-related chromosomes at the margin of a sex- or mating-type locus due to linkage disequilibrium and that such decrease in sequence similarity reduces recombination rates, which further decreases sequence identity as a self-reinforcing process (Jeffries *et al.* 2021). These later hypotheses correspond to both evolutionary and proximal causes of recombination suppression.

The ascomycete fungus *Podospora anserina* is an excellent model for investigating the questions of the evolutionary and proximal causes of recombination suppression. Early genetic analyses showed a peculiar pattern of recombination on the mating-type bearing chromosome (chromosome 1) (Marcou *et al.* 1979), indicating second-division segregation of the mating-type (*MAT*) locus due to an obligate crossover between the *MAT* locus and the centromere of chromosome 1. Several markers were found to be tightly linked to the *MAT* locus, suggesting the existence of a nonrecombining region. Later, a 0.8-Mbp nonrecombining region around the mating-type locus has been described (Grognet, Bidard, *et al.* 2014). There can, however, be some very rare events of recombination in this region (Contamine *et al.* 1996). The nonrecombining region is collinear between the 2 mating types (Grognet, Bidard, *et al.* 2014), indicating that the proximal causes for the lack of crossing overs are not inversions or other genomic rearrangements. A localized hotspot of concentrated repeats within the *MAT*-proximal region was suspected to play a role, but its deletion did not restore recombination (Grognet, Bidard, *et al.* 2014). Regarding the evolutionary cause of recombination suppression, sexual antagonism cannot apply as the mating-type chromosomes do not control any differences in gamete size or behavior (Silar 2020). Furthermore, the haploid phase, in which cells are of alternative mating types, is virtually nonexistent in *P. anserina*, as ca. 99% of sexual spore produced after meiosis are already carrying 2 nuclei of opposite mating types (Marcou *et al.* 1979), a feature that is faithfully maintained in the mycelium (Grognet, Bidard, *et al.* 2014). There is therefore little room in the life cycle for antagonistic selection between mating types. *P. anserina* is mostly selfing, by automixis, so that strains are typically highly homozygous, except in the nonrecombining region around the mating-type locus, that displays differentiation between mating types (98.44% identity between the 2 nonrecombining haplotypes) (Grognet, Bidard, *et al.* 2014; Hartmann, Ament-Velásquez *et al.* 2021). Here, we generated a mutant to test the “neutral hypothesis” postulating that divergence alone is responsible for recombination cessation in the *MAT*-proximal region (Jeffries *et al.* 2021). We replaced the *mat*− sequence at the *MAT* locus by the sequence of the *mat*+ idiomorph, to obtain a *mat+* strain isogenic to the *mat−* strain in the nonrecombining *MAT*-proximal region. We crossed the *mat*+ mutant with the *mat*− wild-type strain, obtaining a dikaryotic strain homozygous in the *MAT*-proximal region. We used strains with resistance genes as markers to detect recombination events. We analyzed the progenies of several crosses, which showed that recombination was still suppressed in the *MAT*-proximal region in the strain homozygous in this region. These findings indicate that other proximal mechanisms than divergence is responsible for recombination suppression in this fungus.

## Material and methods

### Strains and media

**Fig. 1.**
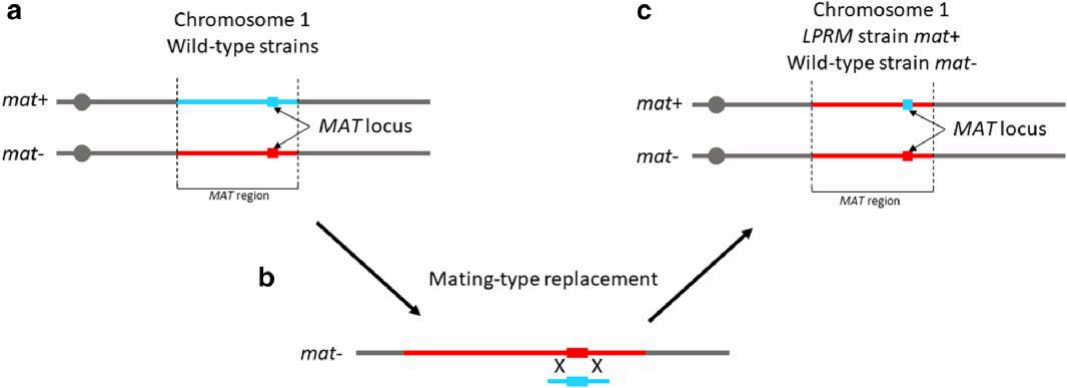
Strategy for the mating-type locus replacement: a) The right arm of chromosome 1 for both *mat*+ and *mat*− wild-type strain is depicted (not to scale). The *MAT* region is the 0.8-Mbp region devoid of recombination. The squares represent the *MAT* loci containing the mating-type genes. b) The *mat*− strain has been transformed with a linearized plasmid containing the *mat*+ sequence and a plasmid containing the hygromycin resistance marker. This latter plasmid was targeted to a gene previously identified as an optimal target for genomic integration of any DNA fragment (Déquard-Chablat *et al*. 2012). Thanks to 2 recombination events, the *MAT* locus was replaced, generating the *LPRM* (Locus Plus-Region Minus) strain. c) The *LPRM* strain can be crossed with a wild-type *mat*− strain. In this context, the *MAT* regions are strictly identical, except for the *MAT* locus itself.

All strains used in this study were derived from the wild-type *S* stain (Rizet and Engelmann 1949; Espagne *et al*. 2008). The strain used for transformation was deleted for *ku70* (El-Khoury *et al.* 2008). In that strain, the *ku70* gene was replaced by a geneticin resistance cassette. The transformation performed as previously described (Brygoo and Debuchy 1985; Silar 2020). The other strains are described in Supplementary Table 1. The media composition can be found in Silar (2020). The strategy for the generation of the mutant strain is described in the *Result* section and in Fig. 1 and Supplementary Fig. 1.

### Plasmids

The plasmid containing the *mat*+ locus sequence comes from the *P. anserina* 10-kbp plasmid genomic library (Espagne *et al.* 2008). Plasmid GAOAB40BC11 contains the *mat*+ locus (3,842 bp) with 2.9 kbp upstream and 3.9 kbp downstream. The plasmid was linearized by *Nar*I before transformation. *Nar*I is absent from the *P. anserina* sequences cloned in GAOAB40BC11 and present in the vector sequences of this plasmid.

Since the transformation is made in a Non-Homologous End Joining (NHEJ)-defective strain (*i.e*. unable of nonhomologous end joining) (El-Khoury *et al.* 2008), the plasmid containing the hygromycin resistance cassette used for co-transformation needs to integrate via homologous recombination; therefore, a part of the plasmid sequence should display similarity with the *P. anserina* genome to target the integration. The targeted locus must be on a chromosome different from the mating-type chromosome to allow markerless replacement of the *MAT* locus (Supplementary Fig. 1). We chose to target the integration in the coding sequence of the *Pa_2_3690* gene, so we cloned its sequence into the pBC-Hygro plasmid (Silar 1995). The gene *Pa_2_3690*, which encodes a putative protein of unknown function with no conserved domain, is genetically independent from the *MAT* locus, its inactivation does not lead to abnormal phenotype (Ait Benkhali *et al.* 2013), and it has already been used for targeted integration (Déquard-Chablat *et al.* 2012). The *Pa_2_3690* coding sequence (1,935 bp) was PCR-amplified using primers pBCpro3690 (ggccgctctagaactagtggatcccccTCACTCCACAAGGCAGCATCTAA) and pBCter3690 (aagcttgatatcgaattcctgcagcccCAGGTGCAAAGGTAACACTCGGT). The purified PCR product was cloned into pBC-Hygro linearized by *Sma*I using NEB's NEBuilder HiFi DNA Assembly to give plasmid pBCH3690.

### Transformation

Three transformations were performed, yielding 18, 33, and 158 hygromycin-resistant transformants, respectively. The mating-type phenotypes of the hygromycin-resistant transformants were tested by confronting each of them with tester strains of known mating types. From the first transformation, 1 transformant was able to mate only with the *mat*− tester strain, indicating that its mycelium contained only *mat*+ nuclei and therefore that the replacement of the mating-type idiomorph was successful. Another transformant could mate with both tester strains, suggesting that it contained *mat*+ and *mat*− nuclei; a mixture of *mat+* and *mat*− nuclei is not surprising as *P. anserina*'s protoplasts used for transformation can contain several nuclei. The remaining 16 transformants mated only with the *mat+* tester strain, indicating transformation with only the hygromycin resistance cassette but not the mating-type idiomorph. The 2 other transformation attempts gave, respectively, 0 and 6 *mat*+ transformants, 3 and 109 transformants with both mating types, and 30 and 39 *mat*− transformants. From the last transformation assay, 4 transformants were not able to mate with any of the tester strains. The following steps are described in the *Result* section.

### Sequencing

Amplifications of sequences upstream and downstream of the *mat+* idiomorph of the *LPRM* (Locus Plus–Region Minus) strain were performed with the 2 primer pairs gggacctctgcagggaat/cactggaacggaggagga and tgacgaatgaaatcgtcgaa/gacccaccgaacctcctc. The genome of strain *LPRM* was sequenced using Illumina technology (paired-end 150 bp at Novogene). The reads were mapped on both *mat+* and *mat−* genome sequences using bowtie2 (version 2.5.1). Mutations were called using samtools mpileup (version 1.9) and processed with custom-made R scripts. Around the mating-type locus, the sequence was confirmed by visual inspection of the mapped reads.

Surprisingly, 3 small possible deletions (170, 55, and 240 bp long, respectively) were detected on chromosome 2 compared with the reference genome published for the *S* strain (Espagne *et al.* 2008). The 2 first ones were in the coding sequences of putative genes: *Pa_2_1350*, which has no predicted domain and is present only in *Podospora* species or close relatives, and *Pa_2_3445*, which has no predicted function but carries a kinesin domain and is conserved in ascomycete fungi. None of these 2 genes seem to be expressed in the available RNA-seq data (Lamacchia *et al.* 2016; Benocci *et al.* 2018; Silar *et al.* 2019; Lelandais *et al.* 2022). The third small deletion was in an intergenic region between the genes *Pa_2_6390* and *Pa_2_6380*. To understand these deletions, we checked the aligned reads of previous sequencing data of our wild-type *S* strain (ChIP-seq data, Carlier *et al.* 2021). These 3 putative deleted sequences were also devoid of aligned reads in these samples (Supplementary Fig. 2), suggesting errors in the reference genome sequence or that these mutations occurred in our wild-type *S* strain prior to the experiments performed here. The rest of the genome does not show any other mutation, except a few C:G/T:A substitutions on repeated sequences that might be caused by RIP (repeat-induced point mutations (Gladyshev 2017)).

### Statistical analyses

The 2 × 2 contingency tests were performed with the GraphPad software (https://www.graphpad.com/quickcalcs/contingency1), with Fisher's exact tests for small sample size and 2-tailed *P*-values.

## Results and discussion

### Mating-type locus replacement

In order to get sexually compatible strains with isogenic *MAT*-proximal regions, we replaced the *MAT* locus of a *mat*− strain by the *mat*+ idiomorph. To do so, a linearized plasmid containing the *mat*+ idiomorph sequence was co-transformed with a plasmid containing a hygromycin resistance cassette in a NHEJ-defective strain (*i.e*. unable of non-homologous end joining) (El-Khoury *et al.* 2008), to allow only integration by homologous recombination at the mating-type locus. Integration of the *mat*+ containing plasmid thanks to 2 recombination events leads to the replacement of the *MAT* locus (Fig. 1 and Supplementary Fig. 1). The hygromycin resistance cassette allows the selection of the transformant, as co-transformation is efficient in *P. anserina*: a plasmid containing a resistance gene is mixed with the replacement cassette (five to ten times more of the cassette) prior transformation. In such conditions, most hygromycin-resistant transformants will here be expected to have also integrated the cassette at the *MAT* locus, which can then be checked. The plasmid containing the hygromycin resistance marker was targeted to the gene *Pa_2_3690*, which was previously identified as an optimal target for genomic integration of any DNA fragment (Déquard-Chablat *et al.* 2012). Three transformations were performed, yielding 209 hygromycin-resistant transformants in total. The mating-type phenotypes of the hygromycin-resistant transformants were tested by confronting each of them with tester strains of known mating types. From 3 independent transformations, 7 transformants were able to mate only with the *mat*− tester strain, indicating that their mycelium contained only *mat*+ nuclei and therefore that the replacement of the mating-type idiomorph was successful.

Five of the *mat*+ transformants from the different transformation assays were crossed with the *mat*− wild-type strain, and homokaryotic spores from the progenies were isolated. Subsequent crossing of these progenies allowed us to recover a NHEJ-proficient strain without the hygromycin resistance cassette harbored by the plasmid used for co-transformation. Hence, the *mat*+ individuals from these progenies carry the *mat*+ idiomorph sequence but the rest of the genome should be identical to the *mat*− strain, including in the *MAT*-proximal region. One of these strains, from now on called *LPRM* (Locus Plus-Region Minus) (Fig. 1), was randomly selected and used for further investigation.

### Sequence analyses of the mutant strain

**Fig. 2.**
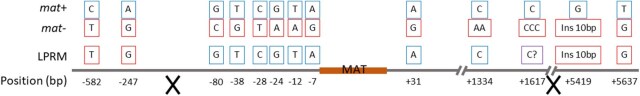
Sequence polymorphism analysis around the *MAT* locus. Base pairs specific to *mat*+ (blue) and *mat*− (red) are shown on top and their position relative to the *mat*+ idiomorph at the bottom. The sequence of the *LPRM* strain carries *mat+* SNPs until 80 bp downstream of the *mat+* idiomorph and *mat*− SNPs further away. On the other side, *mat*+ SNPs are found until 1,617 bp upstream of the *mat+* idiomorph and *mat*− SNPs and indels are present from the next polymorphism at 5,419 bp. The crosses indicate the localization of the recombination events. Ins, insertion.

In order to have a first confirmation of the replacement of the *MAT* locus and to localize the recombination events, we first PCR-amplified the regions directly adjacent to the mating-type locus on both sides and sequenced the 800-bp PCR amplicons. We searched within these sequences for single nucleotide polymorphisms (SNPs) and indels previously identified between the wild-type *mat*+ and *mat*− strains (Grognet, Bidard, *et al.* 2014). On the side (toward the centromere), the sequence obtained was identical to the sequence of the *mat*+ strain up to 80 bp away from the *MAT* locus, but had a *mat−* allele at the next SNP, 247 bp away from the *MAT* locus (Fig. 2), indicating that the recombination with the cassette occurred between these 2 positions. On the other side, only 1 SNP was present and it displayed the *mat*+ allele. To further check the locus replacement, we sequenced the whole genome of the *LPRM* strain. We looked at the sequence around the *MAT* locus and focused on SNPs and indels (Fig. 2). The genome sequence confirmed that the recombination events replacing the *MAT* locus took place as expected, on the 1 side between positions −80 and −247 (in bp, relative to the border of the *MAT* locus), as the site at −80 shows a *mat*+ genotype and the site at −247 shows a *mat−* genotype. On the other side, sites at positions +31 and +1,334 both carried a *mat+* genotype, whereas sites at +5,419 and further away were all of *mat−* genotype. The allele of the site at position +1,617 could not be accurately determined due a long stretch of C and a poor read coverage at that position. The rest of the *MAT*-proximal region carried the *mat−* specific base pairs, indicating that the replacement of the *mat−* idiomorph occurred without affecting the chromosomal structure of the *MAT*-proximal region.

### The mutant *LPRM* strain phenotype is similar to the wild type

**Fig. 3.**
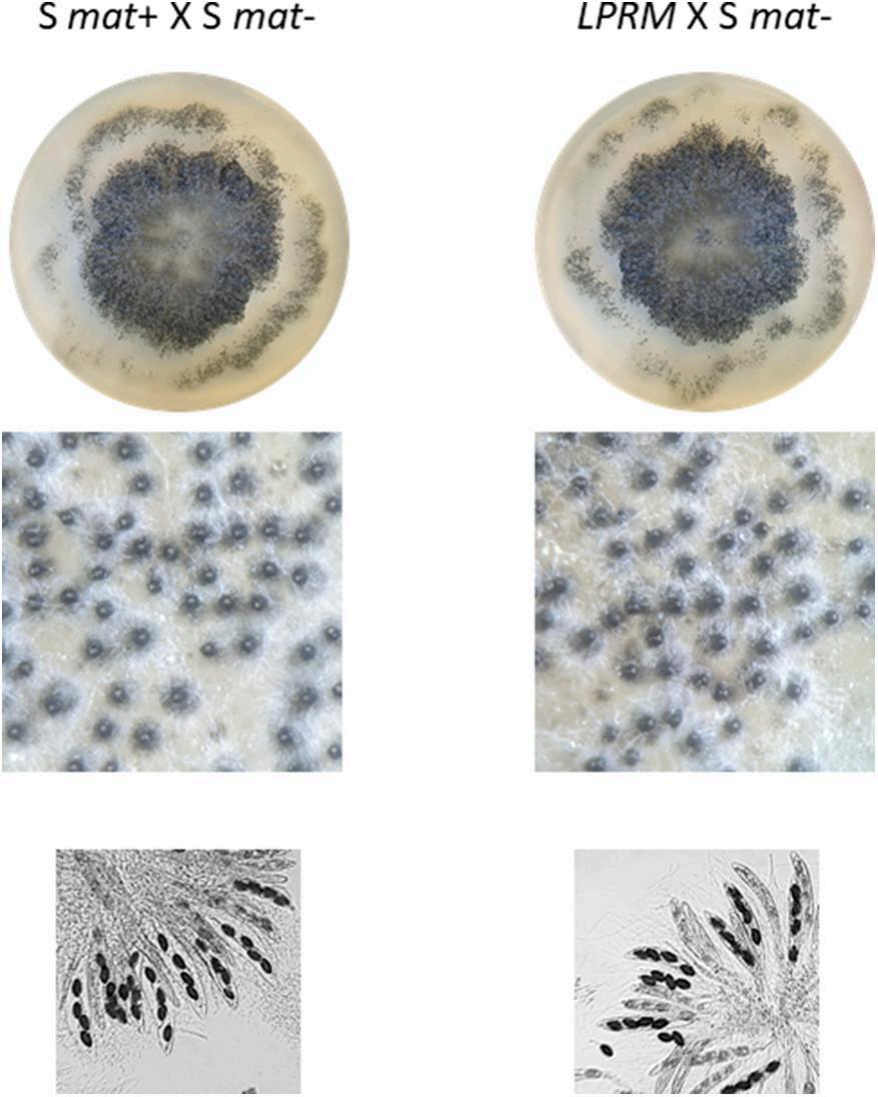
*LPRM* strain phenotype. The *LPRM* strain was compared to the *S mat*+ strain in heterokaryon with the *S mat*− strain. These 2 heterokaryons were indistinguishable in terms of mycelium growth (upper pictures), perithecium formation (middle pictures), or spore formation (lower pictures).

The *LPRM* strain phenotype was indistinguishable from the 1 of the wild-type *S* strain. When grown as monokaryon, the mycelium had the same growth rate, pigmentation, and overall aspect as the *S* strain (data not shown). We also compared the phenotype of a heterokaryon (which is the dominant life stage of *P. anserina*) of the *S* strain (*mat*+ and *mat*−) with a heterokaryon resulting from the vegetative fusion of *LPRM* and *S mat*− (Fig. 3). The 2 heterokaryons were again indistinguishable: they both formed the typical ring of perithecia, the perithecia were equally numerous and properly shaped, and the spores were normally formed and in similar amounts. These observations suggest that, at least in our laboratory conditions, the homozygosity at the *MAT*-proximal region has no obvious effects on vegetative or reproductive traits.

### Crosses indicate suppressed recombination in the mutant strain homozygous in the *MAT*-proximal region

**Fig. 4.**
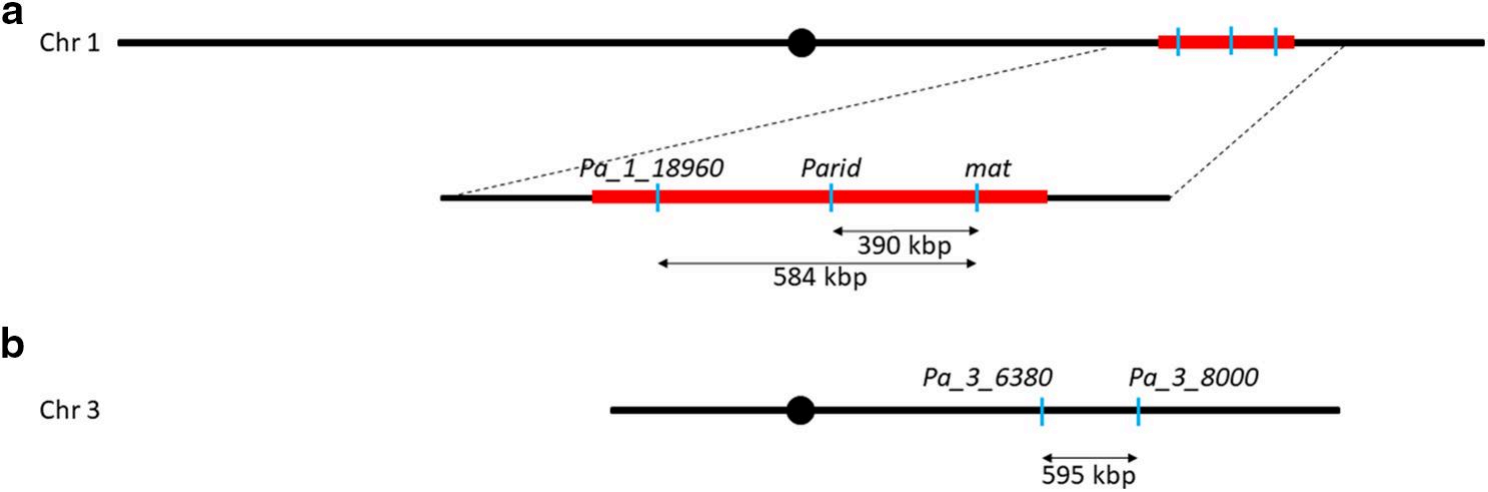
Schematic representation of a) chromosome 1 and b) chromosome 3. Chromosome sizes are scaled as well as the position of the centromere (black disk), the loci of the markers used in the study (blue line), and position and size of the nonrecombining *MAT* region (in red). Physical distances are given below the double-headed arrows.

To test whether the inhibition of recombination still occurs in a cross of *LPRM* with *S mat*− despite the homozygosity in the *MAT*-proximal region, we took advantage of 2 strains already available with hygromycin resistance markers in the *MAT*-proximal region, to detect recombination events (Fig. 4a). The Δ*PaRid* strain (Grognet *et al.* 2019) had the CDS (coding sequence) of the *PaRid* gene replaced by a hygromycin resistance cassette. The *PaRid* gene encodes a putative DNA methyltransferase. The Δ*Pa_1_18960* strain (Grognet, Bidard, *et al.* 2014) had the CDS of the *Pa_1_18960* gene replaced by a hygromycin resistance cassette. The *Pa_1_18960* gene encodes a putative protein with agglutinin-like conserved domain but of unknown function. The distance between *PaRid* and the *MAT* locus is 390 kbp, and the distance between *PaRid* and *Pa_1_18960* is 194 kbp. These 2 strains can be crossed, and previous work showed that there is no recombination between these loci and the *MAT* locus. Hence, when crossing the 2 mutant strains [Hygro^R^, mat−] with *S mat*+ and *LPRM* [Hygro^S^, mat+], recombination events in the *MAT*-proximal region should produce [Hygro^R^, mat+] and [Hygro^S^, mat−] progeny and can thereby be easily detected. We made these 4 crosses twice (each of the 2 mutant strains [Hygro^R^, mat−] with *S mat*+ and *LPRM*) and collected homokaryotic spores from each cross. We looked for recombinants in the homokaryotic progeny by determining (1) the mating type with tester strains and (2) hygromycin resistance on selective medium. The results are given in Table 1. When *S mat*+ was crossed with either Δ*PaRid* or Δ*Pa_1_18960*, no recombinants in the *MAT*-proximal region were recovered across 174 and 188 analyzed offspring, respectively. When *LPRM* was crossed with Δ*PaRid*, no recombinants were recovered from 210 offspring, and when crossed with Δ*Pa_1_18960,* a single recombinant was found out of 222 offspring (0.45%). Given the distance between the *MAT* locus and the 2 other loci, we could expect much more recombination events if the recombination were fully restored (see below).

**Table 1.** Number of offspring showing recombination or no recombination between markers near the mating-type locus and the mating-type locus, in wild type and LPRM contexts.

	*mat−* strains			
	Δ*PaRID mat−*		Δ*Pa_1_18960 mat*−	
*mat+* strains	Number of recombinant ascospores	Number of nonrecombinant ascospores	Number of recombinant ascospores	Number of nonrecombinant ascospores
*S mat+^a^*	0	87	0	92
*S mat+^b^*	0	87	0	96
Total	0	174	0	188
*LPRM mat+^a^*	0	117	1	126
*LPRM mat+^b^*	0	93	0	95
Total	0	210	1	221

^a^Cross # 1.

^b^Cross # 2.

As a point of comparison for the expected number of recombination events given the physical distance, we used 2 other strains, in which the genes *Pa_3_6390* or *Pa_3_8000* have been replaced by a geneticin resistance cassette and a hygromycin resistance cassette, respectively (Grognet, P, unpublished and Debuchy, R, unpublished) (Fig. 4b). The *Pa_3_6390* gene encodes a putative protein with a Udf2p domain (ubiquitin chain elongation factor), and Pa_3_8000 encodes a small putative protein of unknown function. These 2 genes are 595 kbp apart on chromosome 3, which is a similar distance to that between the *MAT* locus and *Pa_1_18960* (used to detect recombination in the cross with the *LPRM* strain). By crossing Δ*Pa_3_6390 mat*− with Δ*Pa_3_8000 mat+,* recombination events between the 2 genes will yield progeny either resistant to both antibiotics or sensitive to both, allowing detecting recombination events. Among the 80 homokaryotic spores tested, we detected 3 recombination events (3.75%; Table 2). Note that the control cross involving the strains Δ*Pa_3_6390* and Δ*Pa_3_8000* was done in a non-*LPRM* background but it would be very unlikely that the modification made in the *LPRM* strain (that involves only the *MAT* region) would affect recombination on the other chromosomes. A cross of *LPRM* with Δ*Pa_1_18960* was made at the same time, and no recombination events between the resistance gene and the *MAT* locus were detected in the 72 homokaryotic spores recovered. Pooling progenies of the 3 identical crosses involving the *LPRM* strain (*LPRM × ΔPa_1_18960 mat*−) indicates that the proportion of recombining offspring in progenies of *LPRM* is significantly different from the proportion in control crosses (a significance threshold of 0.05) (Table 3). A similar percentage of recombinants (3.75%) should indeed have led to ca. 11 recombination events among 294 spores between 2 loci 584 kbp apart on chromosome 1 if recombination had been restored. The results show that the difference in sequences in the *MAT*-proximal region is not responsible for the lack of recombination.

**Table 2.** Number of offspring showing recombination between autosomal markers.

	*mat−* strains			
	Δ*Pa_3_6380 mat−*		Δ*Pa_1_18960 mat−*	
*mat+* strains	Number of recombinant ascospores	Number of nonrecombinant ascospores	Number of recombinant ascospores	Number of nonrecombinant ascospores
*ΔPa_3_8000 mat+*	3	77	ND	ND
*LPRM mat+^a^*	ND	ND	0	72

ND, not determined.

^a^Control cross.

**Table 3.** Statistical comparisons (Fisher's exact test) using a 2 × 2 contingency test between proportions of recombinant progenies in crosses involving the *LPRM* strain and a control cross.

Crosses	Total number of recombinant ascospores	Total number of nonrecombinant ascospores	*P*-value
**Crosses involving the *LPRM* strain**			
*LPRM mat+* x Δ*Pa_1_18960 mat*−	1	293	0.0086
**Control cross**			
Δ*Pa_3_6380 mat*− *×* Δ*Pa_3_8000 mat+*	3	77	N.A

N.A, not applicable.

### What are the molecular mechanisms leading to recombination suppression?

The finding that rendering homozygous the reference strain of *P. anserina* in the *MAT*-proximal region did not restore recombination indicates that sequence divergence alone is not the mechanism responsible for the lack of crossing overs in this region, nor any *cis*-acting recombination modifiers that would need to be heterozygous to act. It also suggests that sequence divergence is rather a consequence than a cause of recombination suppression.

We replaced the *MAT* locus in a *mat−* genomic background, but we did not perform the reversed experiment by introducing the *mat−* idiomorph in the *mat+* genomic background. Therefore, we cannot exclude that whatever triggers recombination suppression is set up by a *mat−* specific allele that would have a dominant effect in a regular cross (*i.e.* with *mat−* and *mat*+ sequences in the *MAT*-proximal region). However, this hypothesis is very unlikely and there is no obvious candidate sequence for such a role identified in this region (Grognet, Bidard, *et al.* 2014).

The *MAT*-proximal region displays 98.44% of sequence identity between the 2 mating types in *P. anserina* (Grognet, Bidard, *et al.* 2014; Hartmann, Ament-Velásquez, *et al.* 2021). In crosses between *Podospora* species showing about the same divergence, recombination occurs normally. For example, *P. anserina* and *Podospora comata* display 98% sequence identity genome wide (Boucher *et al.* 2017; Ament-Velásquez *et al.* 2024), but a cross between these 2 species yields normal recombination rates in the progeny (Espagne *et al.* 2008; Grognet, Lalucque, *et al.* 2014), supporting our conclusion that the sequence divergence observed across the *MAT*-proximal region does not cause recombination suppression.

A single recombination event in the *MAT*-proximal region has been detected in the cross involving the *LPRM* strain. Such a rare event can be expected among a large progeny even in a cross involving strains with wild-type *MAT*-proximal regions (Contamine *et al.* 1996). We have shown that, in a recombination-prone region, the number of recombination events was much higher. In some animal sex chromosomes too, rare events of recombination can occur, as reported for example in frogs (Rodrigues *et al.* 2018). These events have important evolutionary consequences, allowing purging deleterious mutations, regularly “rejuvenating” sex chromosomes (Rodrigues *et al.* 2018).

Because the 2 mating-type chromosomes are collinear (Grognet, Bidard, *et al.* 2014), inversions or other genomic rearrangements are not responsible either for the recombination cessation. Future studies could investigate epigenetic marks, such as methylation and histone modification, to investigate whether the nonrecombining region displays particular patterns that could explain recombination suppression. This would raise the question of what targets the chromatin modification specifically to that region.

This study shows the assets of fungi for testing hypotheses on sex-related chromosomes, being experimentally tractable organisms. Of course, the findings on *P. anserina* do not exclude that neutral divergence can cause recombination suppression in other organisms, but it shows that other causes than mere divergence or inversions can suppress recombination. This conclusion is in agreement with previous experiments which demonstrated that even collinear mating-type chromosomes do not recombine in *N*e*urospora tetrasperma* (Jacobson 2005). In *Microbotryum* fungi, young regions without recombination on mating-type chromosomes can also be collinear and with low levels of divergence (Branco *et al.* 2017). Taken together, these experiments emphasize the role of *trans*-acting inhibitory factors, and tone down the role of chromosome inversions and rearrangements in being the initial proximal cause of recombination suppression, at least in fungi.

The mechanism of recombination suppression and the identification of the *trans*-acting inhibitory factors remain elusive, except in a very few species. Recent progress has been done in the yeast *Lachancea kluyveri* and the green alga *Chlamydomonas reinhardtii*. In *L. kluyveri*, the absence of recombination in a region of 1 Mb encompassing the mating types was correlated with the absence of synaptonemal complex and meiotic proteins required for recombination (Legrand *et al.* 2024). However, whether this absence relies on the presence of an inhibitory factor or the absence of an activating factor is unknown yet. Interestingly, early scientific observations of *P. anserina* meiosis by electron microscopy showed that the synapsis is not complete in the chromosome 1 arm bearing the *MAT* locus (Denise Zickler, personal communication), suggesting that a similar mechanism might be at play in *P. anserina* recombination suppression. DNA cytosine methylation was shown to suppress meiotic recombination in the ∼300-kbp sex-determining region of *C. reinhardtii* (Ge *et al.* 2024). The effect of cytosine methylation on the formation of the synaptonemal complex and recruitment of Spo11 has not been investigated. Therefore, commonalities for recombination suppression in sex- or mating-type determining regions are still elusive. In *P. anserina*, no cytosine methylation was detected on DNA extracted from mycelium (Bewick *et al.* 2019). However, PaRid, a putative *de novo* DNA methyltransferase (not related the *C. reinhardtii* DNMT1), is required for reproduction in this species (Grognet *et al.* 2019). The *PaRid* mutant fails to isolate a pair of *mat+* and *mat−* nuclei from the multinucleated cell into a dikaryotic cell to form the future ascogenous hyphae. A hypothesis that arises from this observation is that a transient DNA methylation, reminiscent of the imprinting phenomenon found in animals, would be required first for nuclei identity and later for the regulation of recombination. Interestingly, genetic analyses showed that, in the rare asci where the *MAT* locus undergoes first-division segregation (instead of second-division segregation), the proportion of double crossovers is higher than what is observed in normal asci and some recombination events in the *MAT*-proximal region can occur (Contamine *et al.* 1996). This observation fits with the hypothesis of an epigenetically based recombination regulation. Under this model, the recombination profile would drastically change and recombination events would be frequent when the regulating epigenetic marks are not properly set up.

### Why did recombination suppression evolve?

Regarding the evolutionary causes of recombination suppression around the mating-type locus, sexually antagonistic selection cannot apply to fungi, as male or female functions are not associated with mating types, and there is little trait associated with mating types (Bazzicalupo *et al.* 2019; Hartmann, Ament-Velásquez, *et al.* 2021). This is especially the case in *P. anserina*, in which any role of mating types in determining male and female functions has been discarded (Grognet, Bidard, *et al.* 2014). In *N. tetrasperma*, a previous study based on differential expression in female and male organs proposed that the nonrecombining haplotypes associated with mating types may control feminization and masculinization (Samils *et al.* 2013), but this hypothesis does not seem supported by phenotypic observations of sexual structure production by alternative mating types (Grognet, Bidard, *et al.* 2014). Furthermore, mating types are expressed at the haploid stage in fungi to control sexual compatibility, and there is virtually no haploid phase in *P. anserina*, during which the 2 mating types could be selected to behave differently, *i.e*. be subjected to sexually antagonistic selection. A hypothesis that can apply to fungi is the selection of nonrecombining fragments that carry fewer deleterious mutations than average in the population, and that are associated with permanently heterozygous loci, which shelter the few deleterious mutations they harbor (Jay *et al*. 2022, 2024). A line of evidence supporting this hypothesis is that recombination is suppressed around the mating-type locus only in fungi having an extended dikaryotic (diploid-like) life stage (Jay *et al.* 2024). In Ascomycetes in particular, recombination suppression around the mating-type locus has repeatedly evolved associated with a prolonged dikaryotic stage, which is consistent with an effect of deleterious mutation sheltering (Menkis *et al.* 2008; Vittorelli *et al.* 2023; Jay *et al.* 2024).

## Supplementary Material

jkaf015_Supplementary_Data

## Data Availability

The *LPRM* genome sequence is available at http://www.ncbi.nlm.nih.gov/bioproject/1191219 (BioProject ID: PRJNA1191219). Plasmids and strains are available upon request. Supplemental material available at G3 online.

## References

[jkaf015-B1] Abbott JK , Nordén AK, Hansson B. 2017. Sex chromosome evolution: historical insights and future perspectives. Proc Biol Sci. 284(1854):20162806. doi:10.1098/rspb.2016.2806. 28469017 PMC5443938

[jkaf015-B2] Ait Benkhali J , Coppin E, Brun S, Peraza-Reyes L, Martin T, Dixelius C, Lazar N, van Tilbeurgh H, Debuchy R. 2013. A network of HMG-box transcription factors regulates sexual cycle in the fungus *Podospora anserina*. PLoS Genet. 9(7):e1003642. doi:10.1371/journal.pgen.1003642. 23935511 PMC3730723

[jkaf015-B3] Ament-Velásquez SL , Vogan AA, Wallerman O, Hartmann FE, Gautier V, Silar P, Giraud T, Johannesson H. 2024. High-quality genome assemblies of 4 members of the *Podospora anserina* species complex. Genome Biol Evol. 16(3):evae034. doi:10.1093/gbe/evae034. 38386982 PMC10936905

[jkaf015-B4] Bachtrog D . 2014. Signs of genomic battles in mouse sex chromosomes. Cell. 159(4):716–718. doi:10.1016/j.cell.2014.10.036. 25417148 PMC4528977

[jkaf015-B5] Bazzicalupo AL , Carpentier F, Otto SP, Giraud T. 2019. Little evidence of antagonistic selection in the evolutionary strata of fungal mating-type chromosomes (*Microbotryum lychnidis-dioicae*). G3 (Bethesda). 9(6):1987–1998. doi:10.1534/g3.119.400242. 31015196 PMC6553529

[jkaf015-B6] Benocci T , de Vries RP, Daly P. 2018. A senescence-delaying pre-culture medium for transcriptomics of *Podospora anserina*. J Microbiol Methods. 146:33–36. doi:10.1016/j.mimet.2018.01.010. 29366759

[jkaf015-B7] Beukeboom L , Perrin N. 2014. The Evolution of Sex Determination. Oxford University Press.

[jkaf015-B8] Bewick AJ , Hofmeister BT, Powers RA, Mondo SJ, Grigoriev IV, James TY, Stajich JE, Schmitz RJ. 2019. Diversity of cytosine methylation across the fungal tree of life. Nat Ecol Evol. 3(3):479–490. doi:10.1038/s41559-019-0810-9. 30778188 PMC6533610

[jkaf015-B9] Boucher C , Nguyen T-S, Silar P. 2017. Species delimitation in the *Podospora anserina/P. pauciseta/P. comata* species complex (Sordariales). Cryptogam Mycol. 38(4):485–506. doi:10.7872/crym/v38.iss4.2017.485.

[jkaf015-B10] Branco S , Badouin H, Rodríguez de la Vega RC, Gouzy J, Carpentier F, Aguileta G, Siguenza S, Brandenburg J-T, Coelho MA, Hood ME, et al 2017. Evolutionary strata on young mating-type chromosomes despite the lack of sexual antagonism. Proc Natl Acad Sci U S A. 114(27):7067–7072. doi:10.1073/pnas.1701658114. 28630332 PMC5502610

[jkaf015-B11] Branco S , Carpentier F, Rodríguez de la Vega RC, Badouin H, Snirc A, Le Prieur S, Coelho MA, de Vienne DM, Hartmann FE, Begerow D, et al 2018. Multiple convergent supergene evolution events in mating-type chromosomes. Nat Commun. 9(1):2000. doi:10.1038/s41467-018-04380-9. 29784936 PMC5962589

[jkaf015-B12] Brygoo Y , Debuchy R. 1985. Transformation by integration in *Podospora anserina*. Mol Gen Genet. 200(1):128–131. doi:10.1007/BF00383325.

[jkaf015-B13] Carlier F , Li M, Maroc L, Debuchy R, Souaid C, Noordermeer D, Grognet P, Malagnac F. 2021. Loss of EZH2-like or SU(VAR)3–9-like proteins causes simultaneous perturbations in H3K27 and H3K9 tri-methylation and associated developmental defects in the fungus *Podospora anserina*. Epigenetics Chromatin. 14(1):22. doi:10.1186/s13072-021-00395-7. 33962663 PMC8105982

[jkaf015-B14] Charlesworth D . 2021. The timing of genetic degeneration of sex chromosomes. Philos Trans R Soc Lond B Biol Sci. 376(1832):20200093. doi:10.1098/rstb.2020.0093. 34247501 PMC8273506

[jkaf015-B15] Charlesworth D , Charlesworth B, Marais G. 2005. Steps in the evolution of heteromorphic sex chromosomes. Heredity (Edinb). 95(2):118–128. doi:10.1038/sj.hdy.6800697. 15931241

[jkaf015-B16] Contamine V , Lecellier G, Belcour L, Picard M. 1996. Premature death in *Podospora anserina*: sporadic accumulation of the deleted mitochondrial genome, translational parameters and innocuity of the mating types. Genetics. 144(2):541–555. doi:10.1093/genetics/144.2.541. 8889519 PMC1207549

[jkaf015-B17] Cortez D , Marin R, Toledo-Flores D, Froidevaux L, Liechti A, Waters PD, Grützner F, Kaessmann H. 2014. Origins and functional evolution of Y chromosomes across mammals. Nature. 508(7497):488–493. doi:10.1038/nature13151. 24759410

[jkaf015-B18] Déquard-Chablat M , Nguyen T-T, Contamine V, Hermann-Le Denmat S, Malagnac F. 2012. Efficient tools to target DNA to *Podospora anserina*. Fungal Genet Rep. 59(1):21–23. doi:10.4148/1941-4765.1011.

[jkaf015-B19] Duhamel M , Carpentier F, Begerow D, Hood ME, Rodríguez de la Vega RC, Giraud T. 2022. Onset and stepwise extensions of recombination suppression are common in mating-type chromosomes of *Microbotryum* anther-smut fungi. J Evol Biol. 35(12):1619–1634. doi:10.1111/jeb.13991. 35271741 PMC10078771

[jkaf015-B20] El-Khoury R , Sellem CH, Coppin E, Boivin A, Maas MFPM, Debuchy R, Sainsard-Chanet A. 2008. Gene deletion and allelic replacement in the filamentous fungus *Podospora anserina*. Curr Genet. 53(4):249–258. doi:10.1007/s00294-008-0180-3. 18265986

[jkaf015-B21] Ellegren H . 2000. Evolution of the avian sex chromosomes and their role in sex determination. Trends Ecol Evol. 15(5):188–192. doi:10.1016/S0169-5347(00)01821-8. 10782132

[jkaf015-B22] Espagne E , Lespinet O, Malagnac F, Da Silva C, Jaillon O, Porcel BM, Couloux A, Aury J-M, Ségurens B, Poulain J, et al 2008. The genome sequence of the model ascomycete fungus *Podospora anserina*. Genome Biol. 9(5):R77. doi:10.1186/gb-2008-9-5-r77. 18460219 PMC2441463

[jkaf015-B23] Fraser JA , Diezmann S, Subaran RL, Allen A, Lengeler KB, Dietrich FS, Heitman J. 2004. Convergent evolution of chromosomal sex-determining regions in the animal and fungal kingdoms. PLoS Biol. 2(12):e384. doi:10.1371/journal.pbio.0020384. 15538538 PMC526376

[jkaf015-B24] Ge T , Gui X, Xu J-X, Xia W, Wang C-H, Yang W, Huang K, Walsh C, Umen JG, Walter J, et al 2024. DNA cytosine methylation suppresses meiotic recombination at the sex-determining region. Sci Adv. 10(41):eadr2345. doi:10.1126/sciadv.adr2345. 39383224 PMC11463267

[jkaf015-B25] Gladyshev E . 2017. Repeat-induced point mutation and other genome defense mechanisms in fungi. Microbiol Spectr. 5(4):10.1128/microbiolspec.funk-0042-2017. doi:10.1128/microbiolspec.FUNK-0042-2017. PMC5607778 28721856

[jkaf015-B26] Grognet P , Bidard F, Kuchly C, Tong LCH, Coppin E, Benkhali JA, Couloux A, Wincker P, Debuchy R, Silar P. 2014. Maintaining two mating types: structure of the mating type locus and its role in heterokaryosis in *Podospora anserina*. Genetics. 197(1):421–432. doi:10.1534/genetics.113.159988. 24558260 PMC4012498

[jkaf015-B27] Grognet P , Lalucque H, Malagnac F, Silar P. 2014. Genes that bias Mendelian segregation. PLoS Genet. 10(5):e1004387. doi:10.1371/journal.pgen.1004387. 24830502 PMC4022471

[jkaf015-B28] Grognet P , Timpano H, Carlier F, Aït-Benkhali J, Berteaux-Lecellier V, Debuchy R, Bidard F, Malagnac F. 2019. A RID-like putative cytosine methyltransferase homologue controls sexual development in the fungus *Podospora anserina*. PLoS Genet. 15(8):e1008086. doi:10.1371/journal.pgen.1008086. 31412020 PMC6709928

[jkaf015-B29] Hartmann FE , Ament-Velásquez SL, Vogan AA, Gautier V, Le Prieur S, Berramdane M, Snirc A, Johannesson H, Grognet P, Malagnac F, et al 2021. Size variation of the nonrecombining region on the mating-type chromosomes in the fungal *Podospora anserina* species complex. Mol Biol Evol. 38(6):2475–2492. doi:10.1093/molbev/msab040. 33555341 PMC8136517

[jkaf015-B30] Hartmann FE , Duhamel M, Carpentier F, Hood ME, Foulongne-Oriol M, Silar P, Malagnac F, Grognet P, Giraud T. 2021. Recombination suppression and evolutionary strata around mating-type loci in fungi: documenting patterns and understanding evolutionary and mechanistic causes. New Phytol. 229(5):2470–2491. doi:10.1111/nph.17039. 33113229 PMC7898863

[jkaf015-B31] Hill WG , Robertson A. 1966. The effect of linkage on limits to artificial selection. Genet Res. 8(3):269–294. doi:10.1017/S0016672300010156. 5980116

[jkaf015-B32] Ironside JE . 2010. No amicable divorce? Challenging the notion that sexual antagonism drives sex chromosome evolution. Bioessays. 32(8):718–726. doi:10.1002/bies.200900124. 20658710

[jkaf015-B33] Jacobson DJ . 2005. Blocked recombination along the mating-type chromosomes of *Neurospora tetrasperma* involves both structural heterozygosity and autosomal genes. Genetics. 171(2):839–843. doi:10.1534/genetics.105.044040. 16020785 PMC1456800

[jkaf015-B34] Jay P , Jeffries D, Hartmann FE, Véber A, Giraud T. 2024. Why do sex chromosomes progressively lose recombination? Trends Genet. 40(7):564–579. doi:10.1016/j.tig.2024.03.005. 38677904

[jkaf015-B35] Jay P , Tezenas E, Véber A, Giraud T. 2022. Sheltering of deleterious mutations explains the stepwise extension of recombination suppression on sex chromosomes and other supergenes. PLoS Biol. 20(7):e3001698. doi:10.1371/journal.pbio.3001698. 35853091 PMC9295944

[jkaf015-B36] Jeffries DL , Gerchen JF, Scharmann M, Pannell JR. 2021. A neutral model for the loss of recombination on sex chromosomes. Philos Trans R Soc Lond B Biol Sci. 376(1832):20200096. doi:10.1098/rstb.2020.0096. 34247504 PMC8273504

[jkaf015-B37] Kent TV , Uzunović J, Wright SI. 2017. Coevolution between transposable elements and recombination. Philos Trans R Soc Lond B Biol Sci. 372(1736):20160458. doi:10.1098/rstb.2016.0458. 29109221 PMC5698620

[jkaf015-B38] Lamacchia M , Dyrka W, Breton A, Saupe SJ, Paoletti M. 2016. Overlapping *Podospora anserina* transcriptional responses to bacterial and fungal non self indicate a multilayered innate immune response. Front Microbiol. 7:471. doi:10.3389/fmicb.2016.00471. 27148175 PMC4835503

[jkaf015-B39] Legrand S , Saifudeen A, Bordelet H, Vernerey J, Guille A, Bignaud A, Thierry A, Acquaviva L, Gaudin M, Sanchez A, et al 2024. Absence of chromosome axis protein recruitment prevents meiotic recombination chromosome-wide in the budding yeast *Lachancea kluyveri*. Proc Natl Acad Sci U S A. 121(12):e2312820121. doi:10.1073/pnas.2312820121. 38478689 PMC10962940

[jkaf015-B40] Lelandais G , Remy D, Malagnac F, Grognet P. 2022. New insights into genome annotation in *Podospora anserina* through re-exploiting multiple RNA-seq data. BMC Genomics. 23(1):859. doi:10.1186/s12864-022-09085-4. 36581831 PMC9801653

[jkaf015-B41] Lenormand T , Roze D. 2022. Y recombination arrest and degeneration in the absence of sexual dimorphism. Science. 375(6581):663–666. doi:10.1126/science.abj1813. 35143289

[jkaf015-B42] Ma W-J , Veltsos P. 2021. The diversity and evolution of sex chromosomes in frogs. Genes (Basel). 12(4):483. doi:10.3390/genes12040483. 33810524 PMC8067296

[jkaf015-B43] Marcou D , Masson A, Simonet J-M, Piquepaille G. 1979. Evidence for non-random spatial distribution of meiotic exchanges in *Podospora anserina*: comparison between linkage groups 1 and 6. Mol Gen Genet. 176(1):67–79. doi:10.1007/BF00334297. 295403

[jkaf015-B44] Menkis A , Jacobson DJ, Gustafsson T, Johannesson H. 2008. The mating-type chromosome in the filamentous ascomycete *Neurospora tetrasperma* represents a model for early evolution of sex chromosomes. PLoS Genet. 4(3):e1000030. doi:10.1371/journal.pgen.1000030. 18369449 PMC2268244

[jkaf015-B45] Muller HJ . 1932. Some genetic aspects of sex. Am Nat. 66(703):118–138. doi:10.1086/280418.

[jkaf015-B46] Ponnikas S , Sigeman H, Abbott JK, Hansson B. 2018. Why do sex chromosomes stop recombining? Trends Genet. 34(7):492–503. doi:10.1016/j.tig.2018.04.001. 29716744

[jkaf015-B47] Rizet G , Engelmann C. 1949. Contribution à l’étude génétique d’un ascomycete tétrasporé: *Podospora anserina*. Rhem Rv Cytol Biol Veg. 11:201–304.

[jkaf015-B48] Rodrigues N , Studer T, Dufresnes C, Perrin N. 2018. Sex-chromosome recombination in common frogs brings water to the fountain-of-youth. Mol Biol Evol. 35(4):942–948. doi:10.1093/molbev/msy008. 29394416

[jkaf015-B49] Samils N , Gioti A, Karlsson M, Sun Y, Kasuga T, Bastiaans E, Wang Z, Li N, Townsend JP, Johannesson H. 2013. Sex-linked transcriptional divergence in the hermaphrodite fungus *Neurospora tetrasperma*. Proc Biol Sci. 280(1764):20130862. doi:10.1098/rspb.2013.0862. 23782882 PMC3712418

[jkaf015-B50] Saunders PA , Muyle A. 2024. Sex chromosome evolution: hallmarks and question marks. Mol Biol Evol. 41(11):msae218. doi:10.1093/molbev/msae218. 39417444 PMC11542634

[jkaf015-B51] Silar P . 1995. Two new easy to use vectors for transformations. Fungal Genet Rep. 42(1):73. doi:10.4148/1941-4765.1353.

[jkaf015-B52] Silar P. 2020. Podospora anserina. HAL. (978-2-9555841-2-5 (hal-02475488)).

[jkaf015-B53] Silar P , Dauget J-M, Gautier V, Grognet P, Chablat M, Hermann-Le Denmat S, Couloux A, Wincker P, Debuchy R. 2019. A gene graveyard in the genome of the fungus *Podospora comata*. Mol Genet Genomics. 294(1):177–190. doi:10.1007/s00438-018-1497-3. 30288581

[jkaf015-B54] Vittorelli N , Rodríguez de la Vega RC, Snirc A, Levert E, Gautier V, Lalanne C, De Filippo E, Gladieux P, Guillou S, Zhang Y, et al 2023. Stepwise recombination suppression around the mating-type locus in an ascomycete fungus with self-fertile spores. PLoS Genet. 19(2):e1010347. doi:10.1371/journal.pgen.1010347. 36763677 PMC9949647

[jkaf015-B55] Wright AE , Dean R, Zimmer F, Mank JE. 2016. How to make a sex chromosome. Nat Commun. 7(1):12087. doi:10.1038/ncomms12087. 27373494 PMC4932193

