# Supplementary material for: Genetic differentiation in the MAT-proximal region is not sufficient for suppressing recombination in Podospora anserina
Source: G3 (Bethesda). 2025 Jan 24;15(4):jkaf015. doi: 10.1093/g3journal/jkaf015 (PMC12005146; doi:10.1093/g3journal/jkaf015)
Supplement: jkaf015_Supplementary_Data [file jkaf015_supplementary_data.zip › Supplemental_Material_Legends_G3-2025-405667.docx]

Figure S1:

Detailed strategy for the generation of the mutant strain (*LPRM*: Locus Plus - Region Minus). a) A mixture of a linear DNA fragment containing the *mat+* idiomorph and a plasmid conferring hygromycin resistance has been transformed into protoplasts prepared from a ∆*ku70 mat*- strain (*i.e*., unable of non-homologous end joining). b) The genetic events taking place at each step are depicted and the selected genotype and phenotype are given. First, the recipient strain is *mat-* and the *ku70* gene has been replaced by a geneticin resistance cassette. Second, after transformation we selected transformants in which the hygromycin-resistance-conferring plasmid had been integrated. Among these, we screened for *mat+* strains, *i.e*. those in which the mating-type locus had been replaced. Third, we crossed these strains with the wild-type strain and selected the *mat*+ progenies sensitive for both geneticin and hygromycin to recover a *ku70*^+^ allele and segregate away the hygromycin resistance cassette.

Figure S2:

Comparison of read coverage at the three loci with putative deletions. Alignment of reads from *LPRM* whole genome sequencing is shown in the lower track. The two upper tracks show the alignment of reads from two controls which should cover the whole genome (namely Input and Mock) of a previous ChIP-seq experiments (Carlier *et al.* 2021). The three putative deleted sequences are devoid of reads in all experiments.
