# Supplementary figures and images for: Genetic differentiation in the MAT-proximal region is not sufficient for suppressing recombination in Podospora anserina
Source: G3 (Bethesda). 2025 Jan 24;15(4):jkaf015. doi: 10.1093/g3journal/jkaf015 (PMC12005146; doi:10.1093/g3journal/jkaf015)

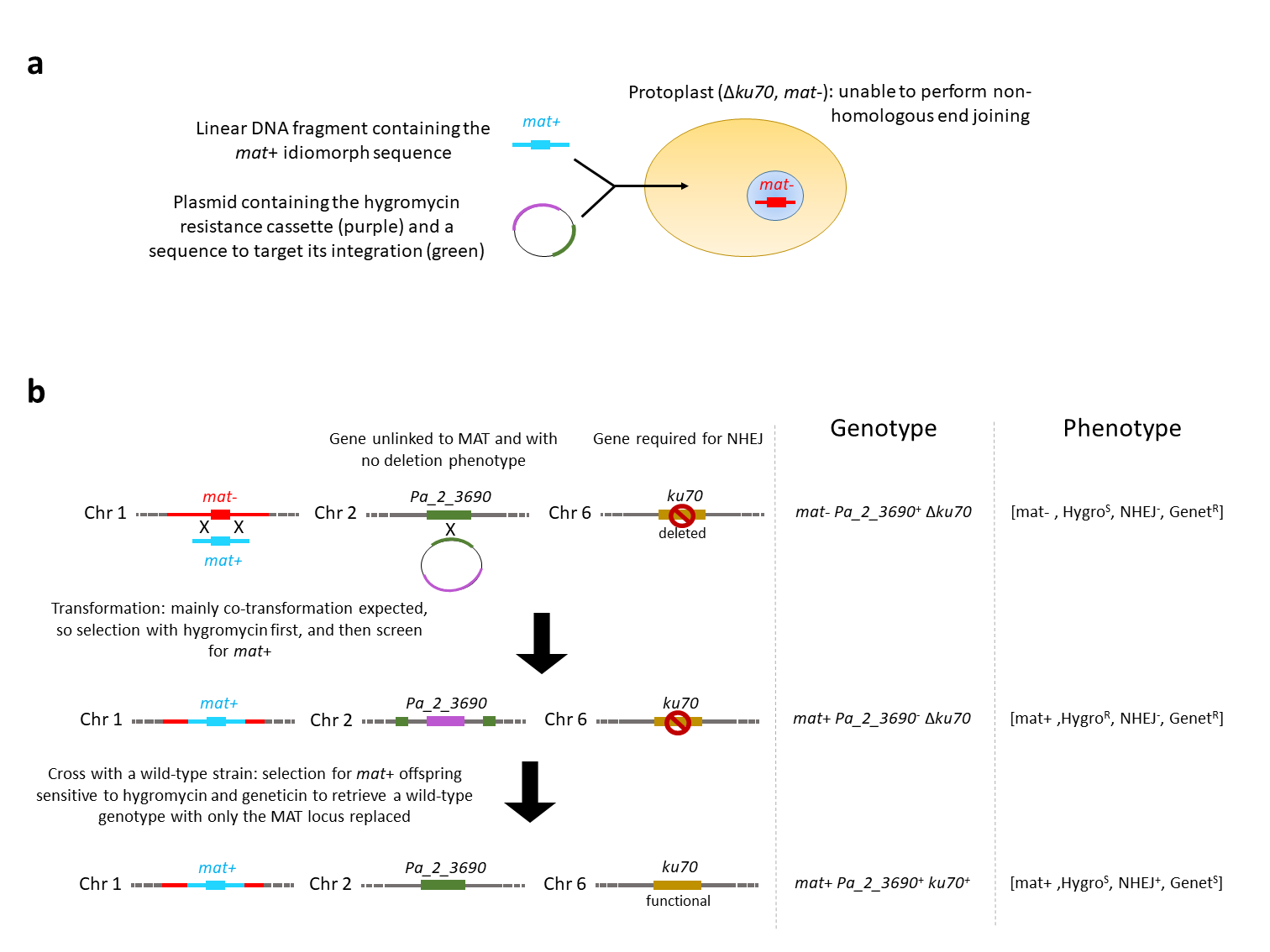

Supplement: jkaf015_Supplementary_Data [file jkaf015_supplementary_data.zip › Figure_S1_G3-2025-405667.tif]

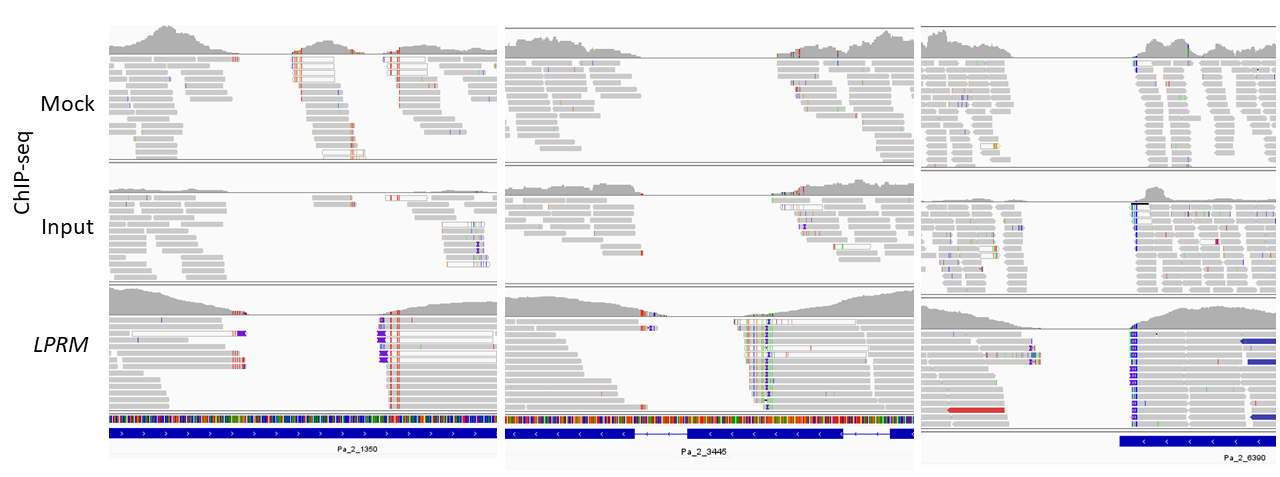

Supplement: jkaf015_Supplementary_Data [file jkaf015_supplementary_data.zip › Figure_S2_G3-2025-405667.tif]
